# Achieving sustained minimal disease activity with methotrexate in early interleukin 23-driven early psoriatic arthritis

**DOI:** 10.1136/rmdopen-2020-001175

**Published:** 2020-07-14

**Authors:** Hannah den Braanker, Kim Wervers, Adriana M C Mus, Priyanka S Bangoer, Nadine Davelaar, Jolanda Luime, Ilja Tchetverikov, J M W Hazes, Marijn Vis, Erik Lubberts, Marc R Kok

**Affiliations:** 1 Department of Rheumatology and Clinical Immunology, Maasstad Hospital, Rotterdam, Netherlands; 2 Department of Rheumatology, Erasmus Medical Centre, Rotterdam, Netherlands; 3 Department of Rheumatology, Albert Schweitzer Ziekenhuis, Dordrecht, Netherlands

**Keywords:** Methotrexate, Psoriatic Arthritis, Cytokines, Epidemiology

## Abstract

**Objectives:**

Methotrexate (MTX) is currently the recommended first-line therapy for treating psoriatic arthritis (PsA), despite lacking clear evidence. No estimates of efficacy of MTX in usual care and no clear MTX responsive clinical or laboratory variables are currently available. This study describes the response to MTX monotherapy in newly diagnosed patients with PsA in usual care. Second, we compared clinical variables and cytokine profiles in patients responding and not responding to MTX monotherapy.

**Methods:**

We used data collected in the Dutch southwest Early Psoriatic Arthritis cohoRt study to select patients with PsA with oligoarthritis or polyarthritis, and at least 1 year follow-up. We analysed disease activity at 6 months of patients who started MTX monotherapy and still used MTX monotherapy 1 year after diagnosis. Cytokine profiles were determined at baseline and after 3 and 6 months with a bead-based multi-immunoassay.

**Results:**

We identified 219 patients of which 183 (84%) patients started MTX monotherapy within 6 months after diagnosis. 90 patients used MTX monotherapy throughout the first year of which 44 patients (24%) reached minimal disease activity(MDA) at 6 months, decreasing to 33 patients (18%) after 1 year. Non-responders had significantly higher concentrations of interleukin (IL) 23 and IL-10 before and during MTX therapy.

**Conclusions:**

Our results showed that only 18% of patients with PsA are in sustained MDA after 1 year of MTX monotherapy and non-responders more often had IL-23-driven disease. Our results indicate the need for more treat-to-target and personalised therapy strategies in PsA.

## INTRODUCTION

Psoriatic arthritis (PsA) is a musculoskeletal disease associated with psoriasis presenting with any or all of peripheral arthritis, axial disease, dactylitis, enthesitis, in addition to skin and nail disease. Inadequately treated, PsA can lead to progressive joint damage and disability. Both current EULAR and Group for Research and Assessment of Psoriasis and Psoriatic Arthritis guidelines advise methotrexate (MTX) as first-line therapy for PsA,^1 2^ despite lacking clear evidence, but due to positive expert experience and limitations of available studies.

Until now, the only randomised controlled trial, the Methotrexate in Psoriatic Arthritis (MIPA) trial,^3^ found no significant effect of MTX monotherapy on several response criteria. However, the MIPA trial did not use the optimal doses of MTX, and its results, therefore, are heavily debated. Furthermore, in the TIght COntrol of inflammation in Psoriatic Arthritis (TICOPA) trial, which compared standard care with treat-to-target therapy strategies for PsA,^4^ only 22.4% of the patients achieved minimal disease activity (MDA) after 12 weeks of MTX monotherapy.^5^ Multiple studies have used MTX monotherapy in their control-arm in randomised controlled trials for tumour necrosis factor inhibitor (TNFi) therapy in PsA or have investigated the MTX response in an observational cohort, with a percentage of patients reaching MDA varying from 18% to 29%.^6–8^ Based on the abovementioned trials, the new American College of Rheumatology/National Psoriasis Foundation guidelines recommend the use of TNFi before MTX^9^ but still classify the evidence as low.^9^

The advantages of MTX in usual care as compared to TNFi are the worldwide availability, the known safety profile and low costs. However, if a large proportion of patients with PsA will not respond to MTX therapy, understanding why and which patients will not respond before start of therapy is needed. Due to the heterogeneous nature of PsA, it might be difficult to identify these patients based on clinical variables alone and adding laboratory variables predictive of MTX response could prove to be valuable.

The current study aims to describe the response to MTX monotherapy early in the disease course of patients with PsA with oligoarthritis or polyarthritis. Second, we aimed to compare cytokine profiles in patients responding and not responding to MTX monotherapy to determine variables that can serve as easy-to-measure therapy-response biomarkers.

## METHODS

### Patients, setting and patient involvement

We used data collected in the Dutch southwest Early Psoriatic Arthritis cohoRt (DEPAR) study, of which details are described elsewhere.^10^ In short, DEPAR collects data to investigate the daily clinical practice of patients with PsA. Patients with a new diagnosis of PsA are eligible to participate if they did not yet receive treatment with disease-modifying antirheumatic drugs (DMARDs) for PsA before the first study visit. Use of DMARDs prescribed for psoriasis was allowed. The baseline visit takes place as soon as possible after diagnosis, within a few weeks. Written informed consent was obtained from all participants according to the Declaration of Helsinki. The study was approved by the local medical research ethics committee of Erasmus Medical Centre Rotterdam, the Netherlands (MEC-2012-549). During the feasibility stage, the research questions and the choice for patient-related outcome measures were informed by discussions with patients. For this analysis, we used data collected between August 2013 and April 2018, of patients with a phenotype of oligoarthritis (2–4 joints involved) or polyarthritis (5 or more joints involved) at baseline as defined by the treating rheumatologist, and at least 1 year follow-up.

### Data collection

Clinical data and blood samples were collected every 3 months in the first year after diagnosis. Trained research nurses collected data of medication use and clinical data, including swollen and tender joint count (SJC 66 and TJC 68 joints, respectively), enthesitis at clinical examination (Leeds Enthesitis Index,^11^ LEI), dactylitis count, physician global Visual Analogue Scale (VAS) and psoriasis (Psoriasis Area and Severity Index,^12^ PASI). Patients filled out questionnaires shortly before or after their visit to the research nurse. In DEPAR, multiple questionnaires are collected to measure the patient-reported activity of the disease and different outcomes. For the current study, we used self-reported symptom duration before diagnosis, the Health Assessment Questionnaire (HAQ), patient global VAS and patient pain VAS. For the patient global VAS, patients were asked how much their life was affected by their PsA.^13^

### MTX response

In patients with a subtype of oligoarthritis or polyarthritis, we determined whether they initiated MTX monotherapy within 6 months after their diagnosis of PsA and baseline visit and if they continuously used MTX monotherapy, used it intermittently (interruption of more than 2 weeks), had another DMARD added or switched to a different DMARD in the first year after diagnosis. We defined responders as those with low disease activity at 6 and 12 months. We defined non-responders as all patients that had to switch after 3 months to another DMARD or if another DMARD was added after 3 months and those with high disease activity at 6 months. Low disease activity was defined according to EULAR definition (not having one tender and swollen joint),^1^ Disease Activity index for PSoriatic Arthritis Low Disease Activity (DAPSA-LDA (SJC66+ TJC68+ VAS Global (cm) + VAS Pain (cm) + CRP ≤14), and MDA (5/7 remission criteria: SJC ≤1, TJC ≤1, LEI ≤1, PASI ≤1, patient global VAS ≤20 mm, patient pain VAS ≤15 mm and HAQ ≤0.5).^14^ Conversely, those who did not have low disease activity in these different definitions were considered to have high disease activity.

### Multiplex cytokine assay

The serum concentrations of several cytokines were determined by a bead-based immunoassay (LEGENDplex (BioLegend, San Diego, USA)) according to manufacturer’s protocol in the serum of selected responders and non-responders to MTX at T0, T3 and T6 (ie, at time of diagnosis and 3 and 6 months after diagnosis). All samples were analysed on the same day to avoid inter-assay variation. We designed a custom panel of cytokines related to disease pathophysiology,^15–19^ or based on existing evidence of involvement in response to MTX therapy.^20–25^ This panel included interferon gamma (IFNᵧ), granulocyte-macrophage colony-stimulating factor (GM-CSF), TNFɑ, interleukin (IL) 9, IL-10, IL-17A, IL-17F, chemokine C-C motif ligand 20 (CCL20), IL-23, IL-22, IL-33 and ular endothelial growth factor (VEGF). Online supplementary table 1 shows the minimal detectable concentration (MDC) of our assay per cytokine and which bead was used for the detection. We defined definite MTX responders as patients who reached MDA at 6 and 12 months or fulfilled both MDA joints criteria at 6 and 12 months on MTX monotherapy, without the need for steroid injections. We defined definite non-responders as patients who were not using MTX monotherapy continuously or were neither in MDA or EULAR definition of low disease activity at 6 months.

**Table 1:** Baseline characteristics of all patients and divided by starting on MTX monotherapy or not

Characteristics	All patients N = 219	Patients starting on MTX therapy N = 183	Patients not starting on MTX therapy N = 36
**Demographics**			
Age (years)	53±14	53±14	51±16
Male (%)	110 (50)	93 (51)	17 (47)
BMI (kg/m^2^)	28.4±5.0	28.4±4.8	28.6±5.8
Symptom duration (self-reported, months)	10 (4–29)	10 (4–27)	6.4 (4.1–84)
**Baseline disease scores**			
Oligoarthritis (%)	134 (61)	108 (59)	26 (72)
Polyarthritis (%)	85 (39)	75 (41)	10 (28)
Tender joint count	5 (2–10)	5 (2–10)	4 (2–10)
Swollen joint count	3 (2–6)	4 (2–6)	2 (1–4.5)
Elevated CRP >10 mg/L	67 (37)	61 (40)	6 (20)
PASI	2.4 (0.6–4.6)	2.5 (0.7–4.7)	1.3 (0.4–4.5)
Pain VAS	48±27	49±26	47±31
Global VAS	47±25	48±25	39±28
LEI >0 (%)	82 (39)	70 (38)	15 (42)
Dactylitis (%)	35 (16)	32 (17)	3 (8)
HAQ total score	0.8 (0.4–1.1)	0.8 (0.4–1.0)	0.6 (0.3–1.1)
**Medication**			
MTX starting dosage (mg), (%)			
7.5		2 (1)	
10		13 (7)	
15		93 (51)	
20		9 (5)	
25		58 (32)	

Results shown as mean±SD, n (%) or median (IQR). Baseline clinical data of one patient missing, questionnaires of five patients are missing.

BMI, body mass index; CRP C reactive protein; HAQ, Health Assessment Questionnaire; LEI, Leeds Enthesitis Index; MTX, methotrexate; PASI, Psoriasis Area and Severity Index; VAS, Visual Analogue Scale.

10.1136/rmdopen-2020-001175.supp1 Supplementary data

### Statistical analysis

We compared baseline characteristics in patients switching and patients continuing MTX monotherapy using χ^2^ tests, Fisher’s exact tests, t-tests and Wilcoxon rank-sum tests as appropriate. LEGENDplex data analysis software (VigeneTech, Évreux, France) and protocol for data analysis were used to determine the concentration of the cytokines in each sample. To analyse the dynamics of the cytokines over time, marginal effects models were used to compare responders to non-responders. The optimal correlation structure was chosen based on the Akaike information criterion, a statistic relating good fit (ie, likelihood) to complexity of the model (ie, the number of parameters).^26^ For outcomes below the detection limit, the detection limit was chosen as value. Sensitivity analyses using 0 and half the detection limit were performed. Heatmap and associated hierarchical clustering of cytokines was generated using R and the R package heatmap.2 (gplots).^27 28^

## RESULTS

Of 219 patients with a phenotype of oligoarthritis or polyarthritis at time of diagnosis, 183 (84%) started with MTX monotherapy within 6 months of their diagnosis. Table 1 shows the baseline characteristics of patients starting on MTX monotherapy and those who did not. Fifty-one patients with a definition of oligoarthritis or polyarthritis at time of diagnosis have been excluded because they did not have at least 1 year follow-up (27 stopped participating, 8 were lost to follow-up, 4 moved, 5 missed their T12 visit, 7 unknown).

Of the 183 patients starting on MTX monotherapy within 6 months, 90 (49%) continued on MTX monotherapy until 12 months after diagnosis. Of the other 93 (51%) patients, 23 used MTX intermittently, 25 switched to a different DMARD (17 because of side-effects, 7 because of insufficient effect, 1 unknown), and in 45 patients, another DMARD was added (online supplementary figure 1). Table 2 shows the baseline characteristics of all patients who did not take MTX continuously and all patients who remained on MTX monotherapy over the 12-month follow-up period. Only the HAQ score significantly differed at baseline between those two groups.

**Table 2:** Baseline characteristics of patients who continued MTX and those who switched

Characteristics	Patients switching medication N = 93	Patients continuing MTX N = 90
**Demographics**		
Age (years)	52±13	54±14
Male (%)	45 (48)	48 (53)
**Baseline disease scores**		
Swollen joint count	4 (2–7)	3 (2–6)
Tender joint count	6 (3–10)	4 (2–9)
Oligoarthritis (%)	58 (62)	50 (56)
Polyarthritis (%)	35 (38)	40 (44)
PASI	2.6 (0.8–4.9)	2.4 (0.6–4.5)
Pain VAS	51±24	46±26
Global VAS	51±27	46±25
LEI >0 (%)	33 (37)	36 (39)
HAQ total score	0.8 (0.6–1.3)*	0.6 (0.3–0.9)
DAPSA	22.7±10.3	20.4±10.6
MDA (%)	2 (2)	6 (7)

Results shown as mean±SD, n (%) or median (IQR). Baseline clinical data of one patient missing, questionnaires of three patients missing.

*p<0.05.

DAPSA, Disease Activity index for PSoriatic Arthritis; HAQ, Health Assessment Questionnaire; LEI, Leeds Enthesitis Index; MDA, minimal disease activity; MTX, methotrexate; PASI, Psoriasis Area and Severity Index; VAS, Visual Analogue Scale.

**Figure 1:**
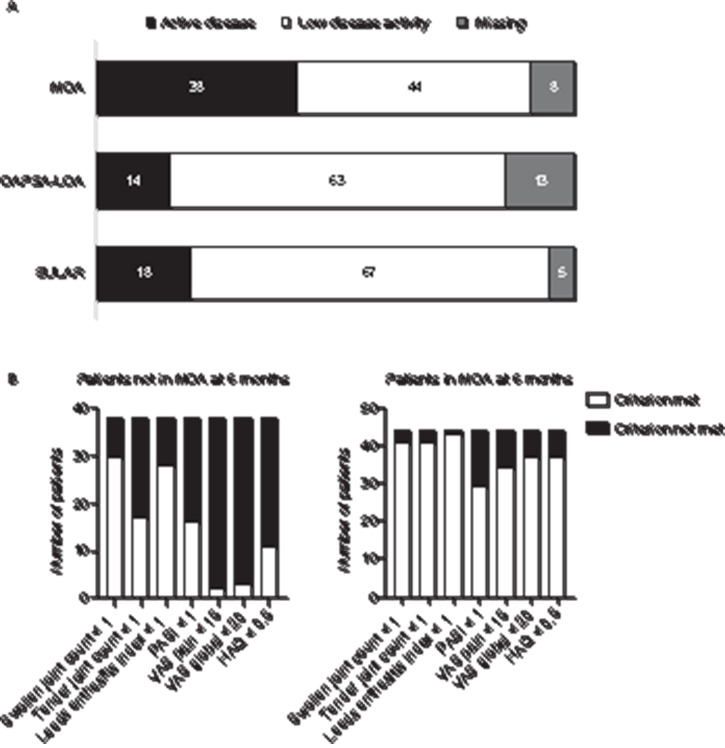
(A) Different bars show the 90 patients using MTX monotherapy continuously in the first year of diagnosis with active disease and low disease activity according to MDA criteria, DAPSA or EULAR definition at 6 months. (B) Graphs show the number of patients that met the MDA criteria included patients in MDA and those that are not in MDA at 6 months. DAPSA, Disease Activity index for PSoriatic Arthritis; MDA, minimal disease activity; MTX, methotrexate.

10.1136/rmdopen-2020-001175.supp2 Supplementary data

### Reaching sustained MDA with MTX monotherapy

Figure 1A shows the 90 patients continuing MTX monotherapy until 12 months meeting the different low disease activity scores. Compared to the other disease activity measures, a lower number of patients of 44 (49%, 24% of total) reached MDA at 6 months. Of these 44 patients, 11 lost their MDA status at 9 or 12 months, and 33 patients reached sustained MDA status for the first year after diagnosis on MTX monotherapy (18% of total). Figure 1B shows which criteria of MDA were met or not by the two different groups. Most patients not in MDA at 6 months did not meet the patient-related outcome measures included in the MDA criteria.

### Longitudinal dynamics of cytokine profiles in the serum of responders and non-responders to MTX monotherapy

**Figure 2:**
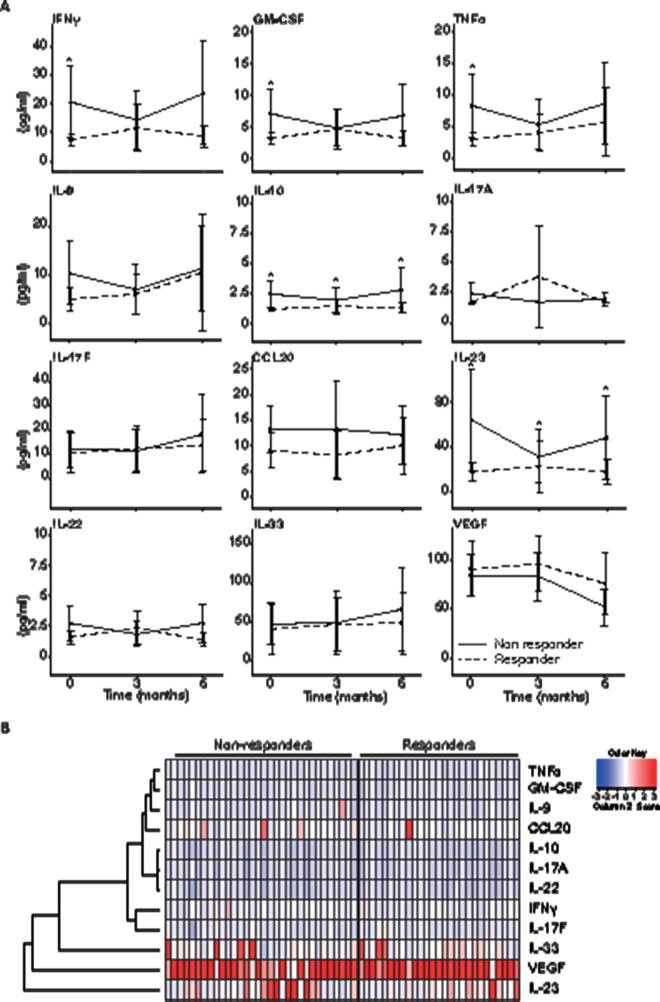
(A) Differences between serum concentrations of 12 cytokines in responders and non-responders analysed using marginal effects models. Non-responders: patients who were not continuously using MTX or were neither in MDA or EULAR definition of low disease activity at 6 months. Responders: patients who reached MDA and EULAR definition of low disease activity at 6 and 12 months on MTX monotherapy. Mean±SD, *p<0.05. (B) Heatmap depicts log2-transformed normalised expression of cytokines (rows) for individual responders and non-responders (columns). Colour code for normalised expression of the cytokines is provided in the key. MDA, minimal disease activity; MTX, methotrexate.

For the analysis of longitudinal dynamics of cytokine profiles, we selected 32 definite non-responders (ie, 83 patients who switched to or added another DMARD after T3 or did not reach MDA and no EULAR remission at T6, minus 28 who switched before T3, minus 5 stopping because of side effects, minus 18 (missing samples)) and 27 definite responders to MTX monotherapy (ie, 48 sustained MDA or sustained joint-criteria, minus 11 patients having had steroid injections and minus 10 patients (missing samples))(see also online supplementary figure 1). At baseline, we found significant higher concentrations of several cytokines in non-responders versus responders, respectively: IL-23 (mean 62.94±25.02 vs 17.44±4.78, p<0.05), TNFα (mean 7.83±2.79 vs 2.63±0.66, p<0.05), GM-CSF (mean 6.5±2.07 vs 2.78±0.64, p<0.05), IFNγ (mean 19.74±6.85 vs 6.67±1.29, p<0.05) and IL-10 (mean 2.08±0.6 vs 1.03±0.13, p<0.05) (figure 2A). In a marginal model analysis, the course of IL-23 and IL-10 over 6 months remained significantly different between responders and non-responders. Figure 2B displays a heatmap of the cytokine profiles in the individual patients. The dendogram shows that IL-23 clusters away from all other cytokines and separates best responders and non-responders to MTX monotherapy at baseline. Clinically, the definite non-responders had at baseline a significantly higher TJC, higher pain VAS, higher global VAS, higher HAQ score, but lower enthesitis score (table 3).

**Table 3:** Difference in clinical characteristics of responders and non-responders at baseline

Characteristics	Non-responders N = 32	Responders N = 27
**Demographics**		
Age (years)	52±14	53±14
Male (%)	20 (63)	17 (63)
**Baseline disease scores**		
Swollen joint count	5 (3–8)	3 (2–5)
Tender joint count	6 (3–10)	4 (2–7)*
PASI	2.6 (1.1–6.5)	1.8 (0.4–3.6)
Pain VAS	54±24	40±30*
Global VAS	57±28	40±24*
LEI >0 (%)	9 (28)	14 (54)*
HAQ total score	0.6 (0.4–1.1)	0.4 (0.1–0.8)*

Results shown as mean±SD, n (%) or median (IQR).

Non-responders: patients who were not continuously using MTX or were neither in MDA or EULAR definition of low disease activity at 6 months. Responders: patients who reached MDA and EULAR definition of low disease activity at 6 and 12 months on MTX monotherapy.

*p<0.05.

HAQ, Health Assessment Questionnaire; LEI, Leeds Enthesitis Index; MDA, minimal disease activity; PASI, Psoriasis Area and Severity Index; VAS, Visual Analogue Scale.

## DISCUSSION

We studied MTX use and response in an observational usual care cohort of patients newly diagnosed with PsA. Half of the patients (49%) who initially presented with oligoarthritis or polyarthritis continued MTX monotherapy until 12 months of follow-up. Only 33 patients of the 183 patients who started on MTX monotherapy (18%) achieved sustained MDA after the first year with MTX monotherapy. We found that non-responders to MTX monotherapy, that is, patients switching to other/additional therapy or not in MDA or EULAR definition of low disease activity at 6 months, had significantly higher baseline concentrations of IL-23, TNFα, GM-CSF, IFN-γ and IL-10.

Our study is the first to assess survival of and response to MTX in usual care, within the first year after diagnosis. Our data reflect daily clinical practice, both concerning the type of patients and the treatment chosen. Treatment was not given in a treat-to-target regimen, such as in the TICOPA study. In the TICOPA study,^5^ 22.4% of patients reached MDA at 12 weeks. Further analysis of efficacy of MTX monotherapy was not possible after 3 months, as treatment was escalated in the tight control group. Other randomised controlled trials including MTX monotherapy as a control group have found comparable outcomes,^6 7^ respectively 29% reaching MDA after 22 weeks, and 22.9% after 24 weeks and the slightly higher 35.7% after 48 weeks. However, the control-arms in these trials of MTX monotherapy do not reflect usual care, as treatment was escalated in many patients and not all patients with PsA were eligible to participate. In another observational cohort including 175 patients with PsA starting on MTX monotherapy for the first time,^8^ only 17.4% of these patients reached MDA at 6 months. However, these patients were not newly diagnosed and patients with concomitant use of other DMARDs were not excluded from this analysis. We studied the survival of MTX in usual care patients and found that 24% was in MDA at 6 months. Disease activity at 6 months was lower according to the less strict definitions of disease activity, that is, the DAPSA-LDA, and EULAR definition. Only 18% could maintain their MDA status up until 1 year after diagnosis, which shows that reaching sustained MDA within the first year after diagnosis on MTX monotherapy is difficult in many newly diagnosed patients with PsA.

Furthermore, our study aimed to screen for easy-to-measure therapy-response biomarkers to aid clinicians in recognising patients that will not respond to MTX monotherapy. We used a sensitive bead-based immunoassay to simultaneously determine concentrations of the 12 cytokines. We found that non-responders to MTX, that is, patients switching to other/additional therapy or not in MDA or EULAR definition of low disease activity at 6 months, had significantly higher baseline concentrations of IL-23, TNFα, GM-CSF, IFNγ and IL-10. Furthermore, our results show that IL-23 was high before, and was not effectively decreased during MTX monotherapy, in the serum of patients not responding to MTX monotherapy. In additional analysis, IL-23 showed to be the best cytokine to differentiate between responders and non-responders to MTX. However, this does depend on this analysis on the higher detectable levels in the serum. Our results correspond with other studies in other diseases treated with MTX monotherapy, such as juvenile idiopathic arthritis, rheumatoid arthritis (RA) or psoriasis, which also found a pre-existent higher concentration of TNFα in non-responders,^20^ or lack of effect of MTX monotherapy on IL-23 or IL-17 serum or plasma concentrations.^29 30^ Together, this may explain why MTX is not effective in patients with PsA with primarily IL-23-driven disease. Surprisingly, we also found consistently higher serum levels of the anti-inflammatory cytokine IL-10 in our non-responders. In RA, IL-10-producing T cells were found to be increased in MTX responders corresponding with the historically viewed anti-inflammatory capacities of IL-10.^31 32^ Our conflicting results might reflect a feedback mechanism of IL-10 in controlling severe disease. However, we found only low levels of IL-10, which might not be biologically relevant.

Not only proinflammatory cytokines were higher at baseline in non-responders than in responders to MTX monotherapy; non-responders also clinically showed higher disease activity, based mainly on the patient-reported measures. Our study shows that IL-23 serum levels can both objectively reflect patient-reported measures and MTX response, making it an interesting therapy-response biomarker.

This study has several strengths and limitations. First, it includes all patients with a new diagnosis of PsA and both the population and the treatment strategy reflect current usual care. It is suitable to describe MTX survival rates in usual care. Drawing conclusions about the effect of MTX should be done with caution, as there could be confounding by indication. Therefore, we chose to display the response on MTX as a percentage of the whole population who started on MTX monotherapy. Second, we used a sensitive assay to compare cytokine profiles in responders and non-responders, not only at baseline but also after 3 and 6 months of MTX therapy to investigate the effect of therapy on all these cytokines. However, we will need to validate the use of serum IL-23 as a therapy-response biomarker in future studies.

In conclusion, our results show that only 18% of patients with PsA achieve sustained MDA with MTX monotherapy after 1 year. Non-responders show worse patient-reported outcome measures and have higher IL-23 serum levels at baseline. This study highlights the potential value of use of new tools, such as measuring cytokine profiles, to stratify patients for underlying driving pathogenic mechanisms of their disease. Subsequently, rheumatologists can use this to personalise therapy choices, not only for MTX, in PsA to improve patient outcomes.

**Key messages**

**What is already known about this subject?**

- MTX is recommended as first-line therapy for articular disease in most PsA treatment guidelines, due to positive expert experience and limitations of available studies demonstrating a lack of significant effect of MTX therapy.

**What does this study add?**

- Only 18% of all patients reach sustained MDA on MTX monotherapy alone in the first year after their diagnosis.
- Patients not responding to MTX monotherapy have high levels of IL-23, which are not lowered by therapy with MTX.

**How might this impact on clinical practice?**

- Personalised therapy strategies are needed in PsA because of the clinical and immunological heterogeneity of disease activity and treatment response.
- IL-23 might serve as therapy-response biomarker.
