# Supplementary material for: Achieving sustained minimal disease activity with methotrexate in early interleukin 23-driven early psoriatic arthritis
Source: RMD Open. 2020 Jul 14;6(2):e001175. doi: 10.1136/rmdopen-2020-001175 (PMC7425114; doi:10.1136/rmdopen-2020-001175)
Supplement: Supplementary data [file rmdopen-2020-001175s001.pdf]

**Supplementary Table 1** Sensitivity of cytokine measurements of bead-based immunoassay

| Cytokine | Bead ID | MDC (pg/ml) |
|---|---|---|
| IFN$_{\gamma}$ | B2 | <4.28 |
| GM-CSF | A6 | <1.89 |
| TNFα | A7 | <1.42 |
| IL-9 | A8 | <2.03 |
| IL-10 | B7 | <0.87 |
| IL-17A | B4 | <1.65 |
| IL-17F | B5 | <1.74 |
| CCL20 | A4 | <2.44 |
| IL-23 | A10 | <5.53 |
| IL-22 | B9 | <0.54 |
| IL-33 | B3 | <9.02 |
| VEGF | B6 | <15.2 |

*Description of cytokines, bead identifiers (bead ID) and minimal detectable concentration (MDC) used in bead-based immunoassay (Legendplex)*
