# Supplementary figures and images for: Achieving sustained minimal disease activity with methotrexate in early interleukin 23-driven early psoriatic arthritis
Source: RMD Open. 2020 Jul 14;6(2):e001175. doi: 10.1136/rmdopen-2020-001175 (PMC7425114; doi:10.1136/rmdopen-2020-001175)

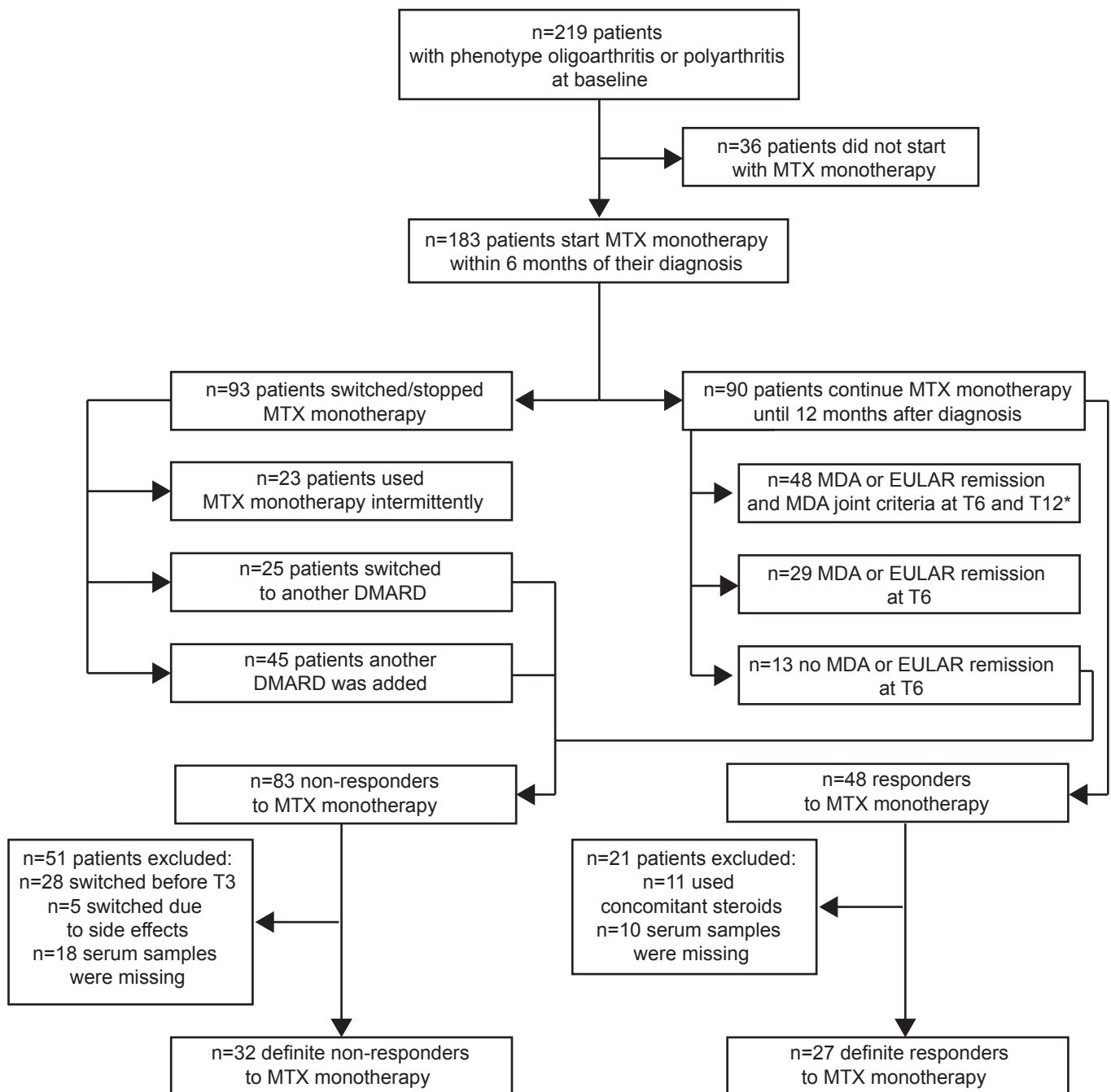

Supplement: Supplementary data [file rmdopen-2020-001175s002.pdf]
